# Lungenschäden durch E‑Zigaretten

**DOI:** 10.1007/s00108-020-00879-w

**Published:** 2020-10-06

**Authors:** B. Mayer, S. Nitschmann

**Affiliations:** 1 Institut für Pharmazeutische Wissenschaften, Karl-Franzens-Universität Graz, Humboldtstraße 46, 8010 Graz, Österreich; 2 Lippetal, Deutschland

## Originalliteratur

Blagev DP, Harris D, Dunn AC et al (2019) Clinical presentation, treatment, and short-term outcomes of lung injury associated with e-cigarettes or vaping: a prospective observational cohort study. Lancet 394:2073–2083

Zwischen März und Oktober 2019 wurden in den USA über 1800 Fälle einer Lungenerkrankung gemeldet, die mit E-Zigaretten (Vaping) in Zusammenhang gebracht wird. Der Zusammenhang zwischen den E-Zigaretten und der Krankheitsursache ist bislang nicht geklärt, jedoch wird eine inhalativ-toxische eher als eine infektiöse Ursache vermutet.

Ziel der im Folgenden vorgestellten Studie war es, klinische Symptomatik, Therapie und Kurzzeitergebnisse in der bislang größten Kohorte von Patienten mit E-Zigaretten-assoziierter Lungenerkrankung („electronic cigarettes or vaping-associated lung injury“ [E-VALI]) zu dokumentieren.

## Fazit

Blagev et al. bezeichnen die Ursache von EVALI als unbekannt. Die Autoren erwähnen THC in einem Nebensatz und ignorieren die bereits im August 2019 bekannte Rolle von Vitamin E. Auch die Warnung vor Aromastoffen in Liquids von E-Zigaretten, die in keinem Zusammenhang mit EVALI stehen, ist unangebracht und trägt zur allgemeinen Verwirrung bei. Blagev et al. beteiligen sich damit an einem Prozess, der als „misinfodemic“ bezeichnet wurde [13]. EVALI wurde weder durch E-Zigaretten noch durch das Dampfen von THC oder CBD verursacht, sondern durch skrupellose Drogendealer, die ihren Kunden Cannabis-Liquids mit bis zu 80 % Vitamin-E-Acetat verkauften.
