# Supplementary material for: Lung injury induced by e-cigarettes
Source: Internist (Berl). 2020 Oct 6;61(11):1204–7. [Article in German] doi: 10.1007/s00108-020-00879-w (PMC7581606; doi:10.1007/s00108-020-00879-w)
Supplement: Related texts

# Research article: Zusammenfassung der Studie

**Authors:** S. Nitschmann

**Affiliations:** Lippetal, Deutschland

[Die Studie wurde methodisch gut durchgeführt, allerdings verfolgten die Autoren einen, wie weitere Untersuchungen ergeben haben, falschen Erklärungsansatz hinsichtlich der Ursache der Lungenerkrankung. Dies wird im Kommentarteil des Beitrags differenziert dargestellt.]

## Studiendesign

- Multizentrische prospektive Kohortenstudie an 13 Kliniken in Utah

### Einschlusskriterien

- Patienten, die zwischen dem 27.06.2019 und dem 04.10.2019 in Utah aufgrund einer Lungensymptomatik bei E-Zigaretten-Konsum eine der Intermountain-Healthcare-Ambulanzen oder Krankenhäuser des integrierten Gesundheitssystems konsultierten
- E-Zigaretten-Konsum mit Exposition gegenüber Nikotin und/oder Tetrahydrocannabinol

### Ausschlusskriterien

- Pneumonie mit Erregernachweis

## Methodik

In die prospektive Kohortenstudie innerhalb von Intermountain Healthcare wurden Patienten eingeschlossen, bei denen eine Lungenerkrankung innerhalb von 90 Tagen nach E-Zigaretten-Konsum aufgetreten war. Alle Patienten wurden einer Task Force, bestehend aus 5 Pneumologen bzw. Intensivfachärzten, zugewiesen. E-Zigaretten-Anamnese, klinische Symptome, Laborparameter, Ergebnisse der mikrobiologischen Tests, Bronchoskopie- sowie Bildgebungsergebnisse, Therapiemaßnahmen, klinischer Verlauf und Follow-up-Daten wurden standardisiert erfasst.

## Ergebnisse

Insgesamt wurden 68 Patienten an die Task Force überwiesen. Acht Patienten wiesen keine E-Zigaretten-assoziierte Lungenerkrankung auf und wurden aus der Studie ausgeschlossen. Zwölf der 60 eingeschlossenen Patienten waren Frauen, das Durchschnittsalter betrug 27 Jahre. Bei 14 der 60 Patienten war anamnestisch ein Asthma bronchiale bekannt.

Die meisten Patienten verwendeten vorgefüllte Patronen oder zumindest fertige Flüssigkeiten. Die längste Zeitspanne des E-Zigaretten-Konsums betrug 5 Jahre, andere hatten erst im letzten Jahr begonnen, E-Zigaretten zu konsumieren, bei einem durchschnittlichen E-Zigaretten-Gesamtkonsum von 225 Tagen. Die Häufigkeit variierte zwischen 1- bis 2-mal pro Woche und „ständig“ bzw. alle 5 min.

Insgesamt 59 der 60 Patienten wiesen pulmonale und 54 gastrointestinale Symptome auf. Eine Hypoxämie (arterielle Sauerstoffsättigung [S_a_O_2_] ≤88 %), Tachypnoe und Tachykardie waren die häufigsten Symptome. Von den 60 erkrankten E-Zigaretten-konsumierenden Patienten wurden 33 auf eine Intensivstation aufgenommen, 10 davon beatmet. In der International Classification of Diseases (ICD) wurden 30 der 60 Patienten mit „akute hyperkapnische respiratorische Insuffizienz“ (J96.01) klassifiziert, weitere ICD-Codes waren unter anderem „akute hypoxische respiratorische Insuffizienz“ (J96.00), „nicht näher bezeichnete Krankheit der Atmungsorgane durch chemische Substanzen, Gase, Rauch und Dämpfe“ (J68.9), „systemisches inflammatorisches Response-Syndrom“ (SIRS; R65.1) sowie das „Atemnotsyndrom des Erwachsenen“ (ARDS; J80).

Eine Leukozytose >11.000/mm^3^ war die häufigste Laboranomalie; C-reaktives Protein (31 mg) und mittlere Erythrozytensedimentationsrate (92 mm/h) waren ebenfalls häufig erhöht.

Behandelt wurden 54 der 60 Patienten mit Antibiotika und 57 mit Steroiden sowie Sauerstoff. Nach Ansicht der behandelnden Ärzte war die Steroidtherapie diejenige Therapie, die zu einer raschen Besserung der Symptomatik führte. Dosis und Dauer der Steroidtherapie variierten.

Sechs der 60 Patienten wurden binnen 2 Wochen erneut in ein Krankenhaus bzw. eine Intensivstation aufgenommen. Drei von ihnen hatten einen Rückfall erlitten. Zwei Patienten wiesen einen Pneumothorax mit nachfolgendem Lungenabszess auf. Der sechste wurde mit Cholezystitis ins Krankenhaus aufgenommen, entwickelte eine hämophagozytische Lymphohistiozytose (HLH) und verstarb im Multiorganversagen.

Insgesamt verstarben 2 Patienten, wobei die E-Zigaretten-Nutzung nicht als Todesursache angesehen wurde: der bereits erwähnte Patient mit HLH und ein weiterer Patient erlitten einen Herzstillstand nach Transkatheteraortenklappenimplantation.

Von 26 Patienten, die über 2 Wochen nachkontrolliert wurden, wiesen 10 von 15 Anomalien im Thoraxröntgenbild und 6 von 9 Patienten eine Einschränkung in Lungenfunktionstests auf. Einer der 26 Patienten konsumierte weiterhin E-Zigaretten.

### Take home message

„Electronic cigarettes or vaping-associated lung injury“ (EVALI) ist die Fehlbezeichnung einer 2019 in den USA ausgebrochenen entzündlichen Lungenerkrankung, die nicht durch die Benutzung von E-Zigaretten, sondern durch den Konsum von illegal mit Vitamin-E-Acetat gestreckten Cannabis-Liquids verursacht wurde.

# Research article: Kommentar

**Authors:** B. Mayer

**Affiliations:** grid.5110.5 0000000121539003 Institut für Pharmazeutische Wissenschaften, Karl-Franzens-Universität Graz, Humboldtstraße 46, 8010 Graz, Österreich

Blagev et al. diskutieren eine Erkrankung, die im Juli 2019 im Grenzgebiet von Illinois und Wisconsin ausbrach und sich bald über die gesamten USA verbreitete [1]. Da nur Nutzer von E-Zigaretten („Dampfer“) erkrankten, bezeichneten die Centers for Disease Control and Prevention (CDC) die Krankheit als „electronic cigarettes or vaping-associated lung injury“ (EVALI). Gemäß abschließender Mitteilung der CDC vom 25. Februar 2020 sind 2807 Menschen mit einem mittleren Alter von 24 Jahren an EVALI erkrankt und 68 daran gestorben [2]. Am 12. August 2019 berichtete das California Department of Public Health, dass alle identifizierten Fälle auf dem Dampfen von Liquids mit Tetrahydrocannabinol (THC) oder Cannabidiol (CBD) beruhten [3], und wenige Tage später veröffentlichte die Plattform Leafly eine Warnung vor „vape pens“ mit illegal gestreckten Cannabis-Liquids [4]. Die CDC publizierten aber monatelang generelle Warnungen vor dem Dampfen, da die Ursache von EVALI angeblich unbekannt sei [5]. Die Food and Drug Administration (FDA) warnte am 4. Oktober vor dem Gebrauch THC-haltiger Liquids, die CDC legten die Ursache erst am 8. November offen [6] und beharren weiterhin auf der Fehlbezeichnung der Erkrankung als EVALI [1]. Durch die verschwommene Berichterstattung hielten die CDC Raucher vom Umstieg auf E-Zigaretten ab und gefährdeten das Leben der Bevölkerung, die über die Gefahren des Konsums THC-haltiger „vape pens“ nicht ausreichend informiert wurde [7]. Prof. Michael Siegel (Boston University School of Public Health), der früher selbst für die CDC tätig war, veröffentlichte in seinem Blog mehr als 20 Artikel zum Thema, denen man weitere Details zu den Verfehlungen der CDC in dieser Causa entnehmen kann [8].

Die Medien verbreiteten die Informationen der CDC unkritisch und berichteten auch bei uns nahezu täglich über „Tote durch E-Zigaretten“, was zu einem massiven Einbruch des Handels und zu Verunsicherung in der Bevölkerung führte. Dass E-Zigaretten seit über 10 Jahren am Markt sind und weltweit von etwa 40 Mio. Menschen ohne nennenswerte Gesundheitsprobleme benutzt werden, blieb ebenso unerwähnt wie die erstaunliche geografische Beschränkung von EVALI auf die USA.

## Tatsächliche Ursache der Erkrankung

In der Lungenflüssigkeit von 94 % der EVALI-Patienten wurde Vitamin-E-Acetat nachgewiesen [9]. Aufgrund einer mittellangen aliphatischen Seitenkette ist Vitamin E lipophil (fettfreundlich). Es ist bekannt, dass Fette bei Inhalation eine lebensbedrohliche Lungenerkrankung (Lipidpneumonie) hervorrufen können, und Vitamin E verursachte in einem Tiermodell EVALI-ähnliche Symptome [6]. Die Biopsien von EVALI-Patienten weisen auf eine akute entzündliche Lungenschädigung hin. Ob es sich um eine Lipidpneumonie handelt, ist aber unklar ([10] mit Korrespondenz). Aufgrund seiner lipophilen Eigenschaften ist Vitamin E nicht zur Verdünnung konventioneller Liquids geeignet, die Substanz ähnelt jedoch in Farbe und Viskosität dem THC-Öl in „vape pens“. Das verleitete US-Drogendealer im Frühjahr 2019 zur lukrativen Geschäftsidee, Cannabis-Liquids mit vergleichsweise günstigem Vitamin-E-Acetat zu strecken [11]. Im Zuge der Verhaftung von mehr als 30 Dealern im Herbst 2019 beschlagnahmte die Polizei kontaminierte „vape pens“ und Grundstoffe zur Herstellung der Liquids im Wert von mehreren Millionen US-Dollar [12].

In ihren Verlautbarungen und Publikationen hatten die CDC darauf hingewiesen, dass zwar Vitamin E als eine Ursache identifiziert worden sei, aber nur 89 % der Betroffenen den Konsum von Cannabis-Liquids zugegeben hätten, sodass andere Ursachen nicht ausgeschlossen werden könnten. Die Ehrlichkeit von Teenagern bezüglich des Gebrauchs von Cannabis darf allerdings bezweifelt werden. Mittlerweile sind die für dieses Desaster verantwortlichen Dealer in Haft, Vitamin E ist vom US-Schwarzmarkt verschwunden und mit ihm auch EVALI.
